# Genome-wide analysis of circRNA regulation during spleen development of Chinese indigenous breed Meishan pigs

**DOI:** 10.1186/s12864-023-09612-x

**Published:** 2023-08-23

**Authors:** Yifu Wang, Jinhua Cheng, Chao Xu, Jian Jin, Wenbin Bao, Shenglong Wu, Zhengchang Wu

**Affiliations:** 1 College of Animal Science and Technology, Yangzhou University, Yangzhou, 225009 China; 2 Institute of Comparative Medicine, College of Veterinary Medicine, Yangzhou University, Yangzhou, 225009 China; 3 International Research Laboratory of Prevention and Control of Important Animal Infectious Diseases and Zoonotic Diseases of Jiangsu Higher Education Institutions, Yangzhou University, Yangzhou, 225009 China; 4 Institute of Animal Science, Jiangsu Academy of Agricultural Sciences, Nanjing, 210014 China

**Keywords:** Porcine, CircRNAs, Spleen, Development

## Abstract

**Background:**

Numerous circular RNAs (circRNAs) have been recently identified in porcine tissues and cell types. Nevertheless, their significance in porcine spleen development is yet unelucidated. Herein, we reported an extensive overlook of circRNA expression profile during spleen development in Meishan pigs.

**Results:**

Overall, 39,641 circRNAs were identified from 6,914 host genes. Among them, many circRNAs are up- or down-regulated at different time points of pig spleen development. Using WGCNA analysis, we revealed two essential modules for protein-coding genes and circRNAs. Subsequent correlation analysis revealed 67 circRNAs/co-expressed genes that participated in immnue-associated networks. Furthermore, a competing endogenous RNA (ceRNA) network analysis of circRNAs revealed that 12 circRNAs modulated *CD226, MBD2, SAMD3, SIT1, SRP14, SYTL3* gene expressions via acting as miRNA sponges. Moreover, the circRNA_21767/miR-202-3p axis regulated *SIT1* expression in a ceRNA manner, which is critical for the immune-based regulation of spleen development in Meishan pigs.

**Conclusions:**

Overall, our results demonstrated that the circRNAs were differentially expressed during different stages of porcine spleen development, meanwhile the circRNAs interacted with immune-related genes in a ceRNA-based fashion. Moreover, we presented biomedical researchers with RNAseqTools, a user-friendly and powerful software for the visualization of transcriptome profile data.

**Supplementary Information:**

The online version contains supplementary material available at 10.1186/s12864-023-09612-x.

## Introduction

Pigs (*Sus scrofa*) are an incredible resource for agriculture and food production, as well as biomedical research for the examination of human development, congenital diseases, and pathogen response networks [1]. Till date, systemic research facilitated the pig industry to critically select for enhanced growth, feed conversion efficiency, and disease resistance. In fact, disease resistant pig breeds like Yorkshire and Landrace experience considerably fewer diseases than the local indigenous pigs in China [2, 3]. The Meishan pigs is one of four popular indigenous breeds in China, with a history spanning over 400 years. It is particularly known for its enhanced fertility, strong immune response, and excellent meat quality. Moreover, some investigations reported that the Chinese native-bred Meishan pigs may possess stronger immunity and disease resistance than the commercial or crossbred pigs [4, 5]. Nevertheless, the genetic basis and regulation mechanism of the immune system are still not fully understood and need to be further explored.

Circular RNA (circRNA) is a newly discovered form of non-coding RNA (ncRNA) known for its enclosed configuration, generated via precursor mRNA back-splicing [6]. In contrast to linear RNAs, circRNAs lack both the 5′ cap and 3′ tail, and are covalently closed, thereby making them more stable, RNase R resistant, and with prolonged half-lives [7]. Given these unique properties, circRNAs are great potential as an optimal biomarker. With increasing advancement in high-throughput RNA-seq technology, a growing number of studies identified circRNA presence and essential roles in various organisms. Emerging evidences revealed that circRNAs critically modulate several physiological processes [8, 9], including immune cells and various immune responses [10, 11]. CircRNAs act in a tissue and developmental stage-specific manner, and multiple reports confirmed their functions in the porcine skeletal muscle [12], embryonic muscle [13], Liver [14], longissimus thoracis [15], brain [16], etc. Unfortunately, studies involving the growth and development of porcine immune organs is incredibly rare. Thus, it is challenging to fully elucidate the modulatory properties of circRNAs in immune cells and immune responses. Hence, herein, we attempted to explore the circRNAs-mediated regulation networks that influence porcine immune organs development.

Spleen is a secondary lymphoid organ involved in older erythrocyte elimination, iron recycle, capture and destruction of pathogens, and induction of adaptive immune responses [17, 18]. Being a critical immunologic organ, there is a strong need to examine spleen development in Meishan pigs. Herein, we systematically identified and characterized circRNAs within the porcine spleen from 8 developmental stages, namely, 1d, 7d, 14d, 21d, 28d, 35d, 120d, and 180d after birth, using an Illumina HiSeq platform. Then, using the weighted gene co-expression (WGCNA) and competing endogenous RNA (ceRNA) interaction network analyses, we demonstrated a critical role of circRNAs during porcine spleen development. Our findings provide a beneficial resource for the circRNAs-mediated modulatory functions in Meishan pigs, as well as annotation of the porcine genome. We also contribute to the enhanced understanding of the mammalian spleen development.

## Results

### Overview of the sequencing information

**Fig. 1:**
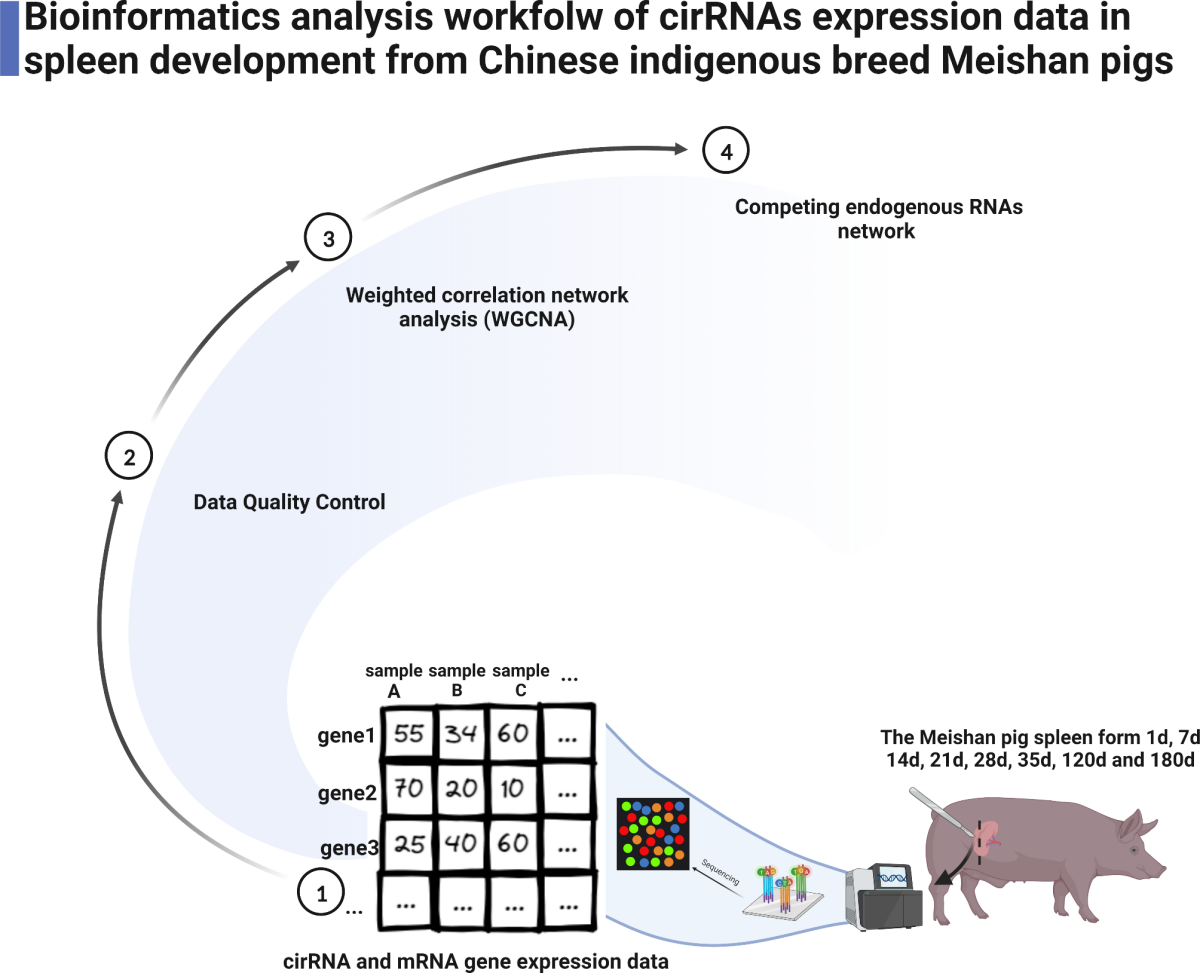
A schematic workflow of circRNAs-mediated regulation of spleen development in the Chinese indigenous breed Meishan pigs. Spleen samples were acquired from 8 distinct developmental stages (1d, 7d, 14d, 21d, 28d, 35d, 120d, and 180d after birth), prior to circRNA sequencing (n = 3 samples per time point), and subsequent downstream analyses

To explore the presence of circRNAs during spleen development, we assessed circRNAs expression in the spleen tissues of Meishan pigs at various developmental stage. We prepared and sequenced ribo-depleted total RNA-seq libraries, as shown in the flow chart (Fig. 1). Table S2 presents our rudimentary sequencing results. At various developmental stages, we acquired around 111.37 million raw reads and 108.68 million clean reads. Additionally, the error rate of all 24 sequencing data sets was below 0.03%, with quality base scores of Q30 > 95%. Together, these data validated the authenticity and precision of our sequencing data.

### Identification and characterization of circRNAs expression in Meishan pigs

**Fig. 2:**
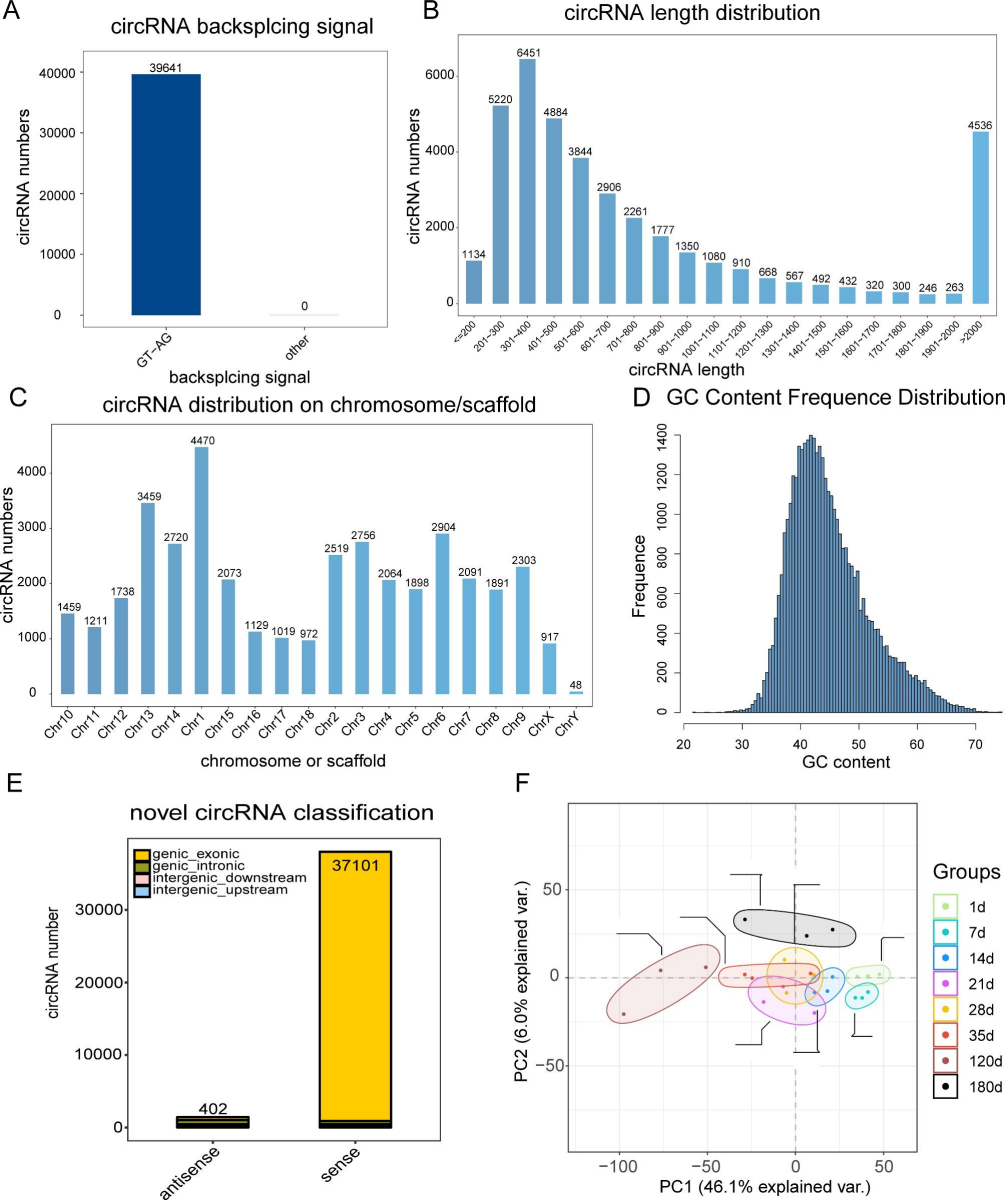
Identification and characterization of circRNAs within Meishan pig spleen. (**A**) circRNAs identification according to the GT-AG backsplicing signal. (**B**) Length allocation of circRNAs. (**C**) Chromosomal allocation of circRNAs. (**D**) GC content frequency allocation of circRNAs. (**E**) circRNAs classification according to the *Sus scrofa* genome. (**F**) Principal component analysis of 24 porcine spleen samples

To characterize the circRNAs in the Meishan pig spleen tissues, we evaluated their sequences. In all, 39,641 candidate circRNAs were identified from 6,914 host genes, using ≥ 1 unique back-spliced read (Fig. 2A). This indicated that most genes potentially produced numerous circRNAs. Their genomic loci showed wide distribution across all chromosomes (Fig. 2B), most (38,507, 97.14%) circRNAs were over 200 nt in length (Fig. 2C), and the GC content of most circRNAs were between 35 and 50% (Fig. 2D). Furthermore, the circRNAs belonged to 3 categories, namely, exonic circRNAs, intronic circRNAs and intergenic. In case of sense circRNAs, most circRNAs (37,101, 97.61%) belonged to the exonic circRNAs category, then the intronic circRNAs category (461, 1.21%), and a small proportion belonged to the intergenic_downstream (225, 0.59%) or intergenic_upstream (223, 0.59%) categories (Fig. 2E). Using principal component analysis (PCA), we revealed that the sample repeatabilities of individual groups were satisfactory (Fig. 2F), confirming that these data can be employed for subsequent DE circRNAs analyses.

### DE circRNAs during spleen development of Meishan pigs

**Fig. 3:**
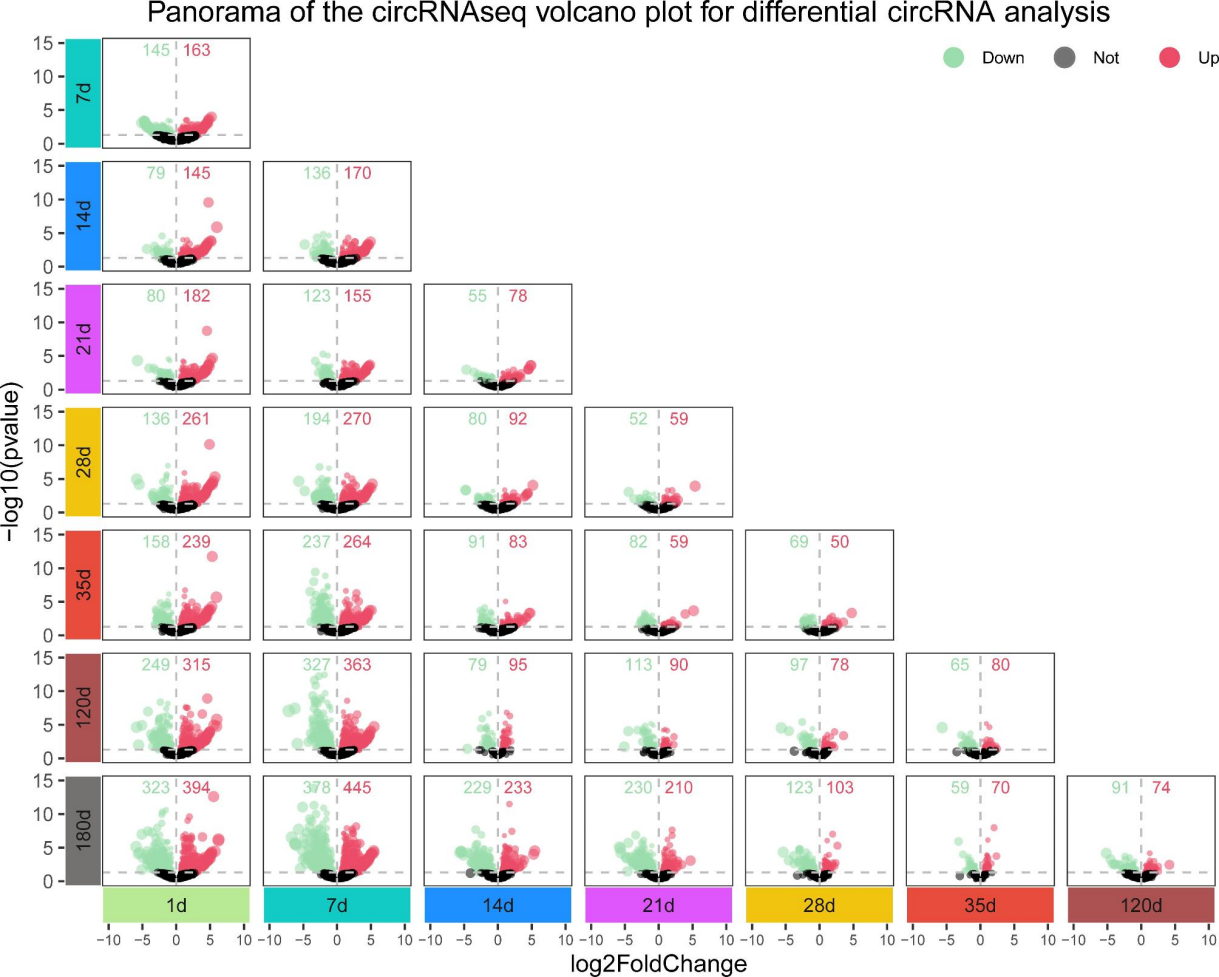
Volcano plot analysis of differentially expressed (DE) circRNAs during different stages of Meishan pig spleen development. Volcano plots depicting –log10 (pvalue) versus log2foldchange in circRNAs expression profile in RPM over different time periods. Red dots indicate highly expressed circRNAs; Green dots indicate scarcely expressed circRNAs.

Emerging evidences support the presence of DE circRNAs during porcine skeletal muscle development [19], however, only limited number of studies explored their temporal expression during spleen development. Herein, we examined the splenic circRNAs expression profiles during different stages of development, namely 1, 7, 14, 21, 28, 35, 120, and 180 days after birth. With 1d (just after birth) as the control, we observed 308, 224, 262, 397, 397, 564, and 717 DE circRNAs during the 7d vs. 1d, 14d vs. 1d, 21d vs. 1d, 28d vs. 1d, 35d vs. 1d, 120d vs. 1d, 180d vs. 1d groups, respectively (Fig. 3). Taken together, these data suggested that the circular nature of circRNAs was potentially critical for its immunomodulatory activity, as multiple circRNAs were either highly expressed or scarcely expressed during distinct stages of porcine spleen development.

### Generation of the circRNA-mRNA co-expression network using WGCNA

**Fig. 4:**
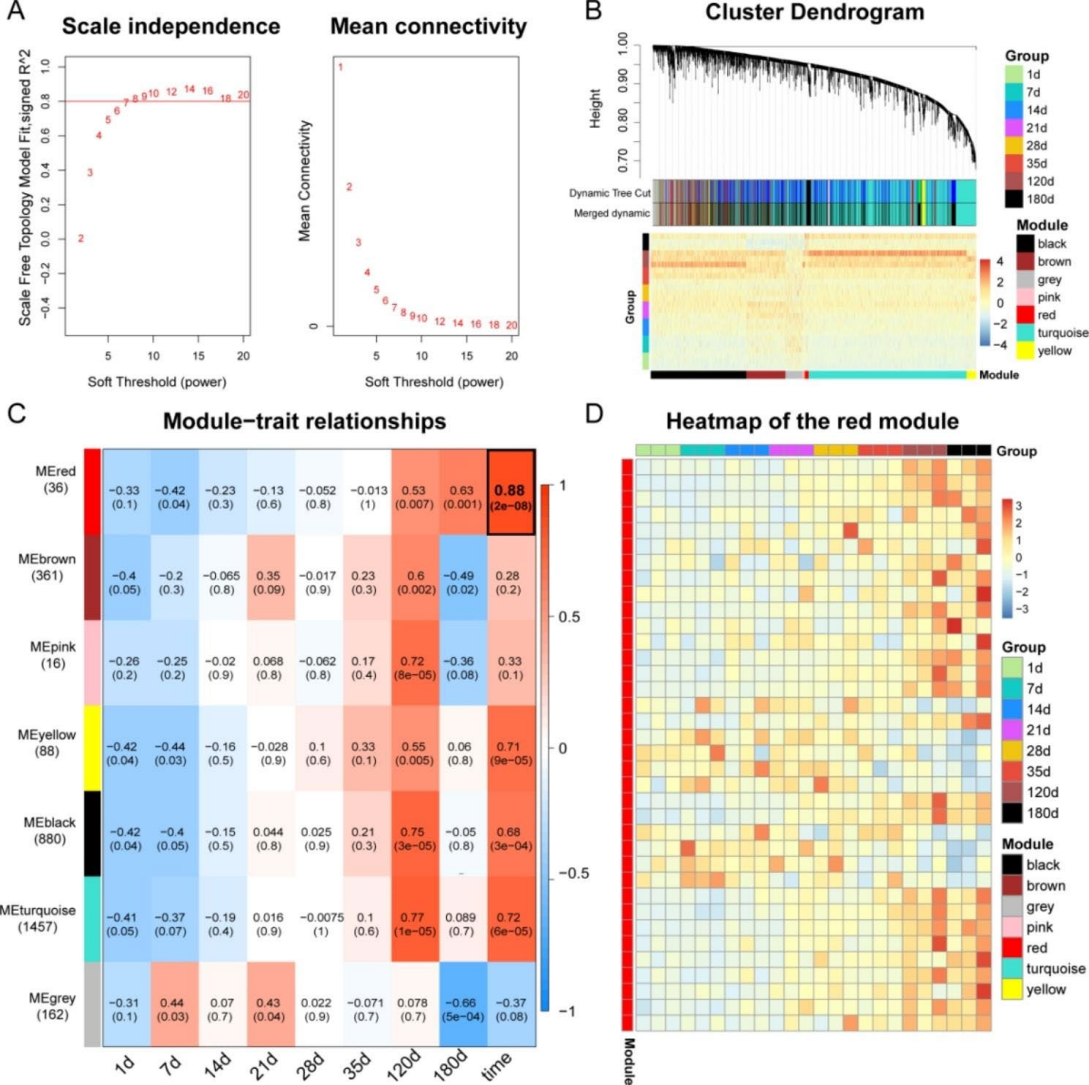
WGCNA analysis of the circRNA dataset identified by the co-expression module of Meishan pig spleen development. (**A**) Scale independence and average connectivity analysis for varying soft cut-off powers. (**B**) Clustering dendrograms of porcine spleen samples at various developmental stages. Differing colors represent WGCNA-generated co-expression modules. Branch modules are color-coded based on their interconnections with other circRNAs. Overall, 7 modules of various colors are presented in the horizontal bar using 0.25 threshold merging. (**C**) The module–trait association. Individual rows represent a modular eigengene, and individual columns represent a trait. Each cell incorporates associated correlation and p-value. Red refers to direct associations, and blue denotes inverse associations. (**D**) Heatmap depicts the circRNA expression profile of the red module

WGCNA is often used to generate gene co-expression modules from gene expression profiles [20]. Herein, we elucidated the circRNAs expression profile by generating a co-expression module containing 3000 circRNAs using WGCNA. With a soft threshold power β of 10 (Fig. 4A), our generated network was categorized into 7 modules, marked by different colors (black, brown, grey, pink, red, turquoise, and yellow) (Fig. 4B, Table S3). Figure 4 C illustrates the association between co-expression modules and various spleen developmental stages. We observed a strong direct correlation between the red modules and developmental stages (correlation coefficient = 0.88, *p* = 2e-08). The heatmap shows the expression pattern of red module, where the expression of genes in the red module tends to increase with time (Fig. 4D). Hence, the red module for circRNA expression profile was selected for subsequent analysis.

We conducted further analysis on a WGCNA module consisting of 15,188 mRNAs, based on a prior transcriptome mRNA profiling of the Meishan pig spleen at distinct developmental stages (NCBI SRA repository under the BioProject IDs: PRJNA875155, PRJNA869328). As depicted in Fig. S1 and Table S4, based on the soft threshold power β = 9 (Fig. S1A), we generated a gene hierarchy clustering tree, and screened 12 gene modules (brown, darkmagenta, darkolivegreen, darkturquoise, sienna3, lightgreen, greenyellow, skyblue, paleturquoise, lightcyan, royalblue, and grey) that were intricately linked to porcine spleen development (Fig. S1B). Additionally, our assessment of the module-trait association revealed that distinct spleen developmental stages were strongly and positively associated with the darkturquoise module (correlation coefficient = 0.74, *p* = 4e-05) (Fig. S1C). Moreover, our heatmap revealed the transcript expression profile of darkturquoise (Fig. S1D). Therefore, we employed the darkturquoise module of mRNAs expression profile for further analyses. Collectively, our circRNA-mRNA co-expression modules revealed distinct expression patterns, and preliminary screening revealed that the circRNAs in the red module and mRNAs in the darkturquoise module displayed a strong direct correlation with spleen developmental stage, thereby confirming a significant role of circRNAs in pig spleen development.

**Fig. 5:**
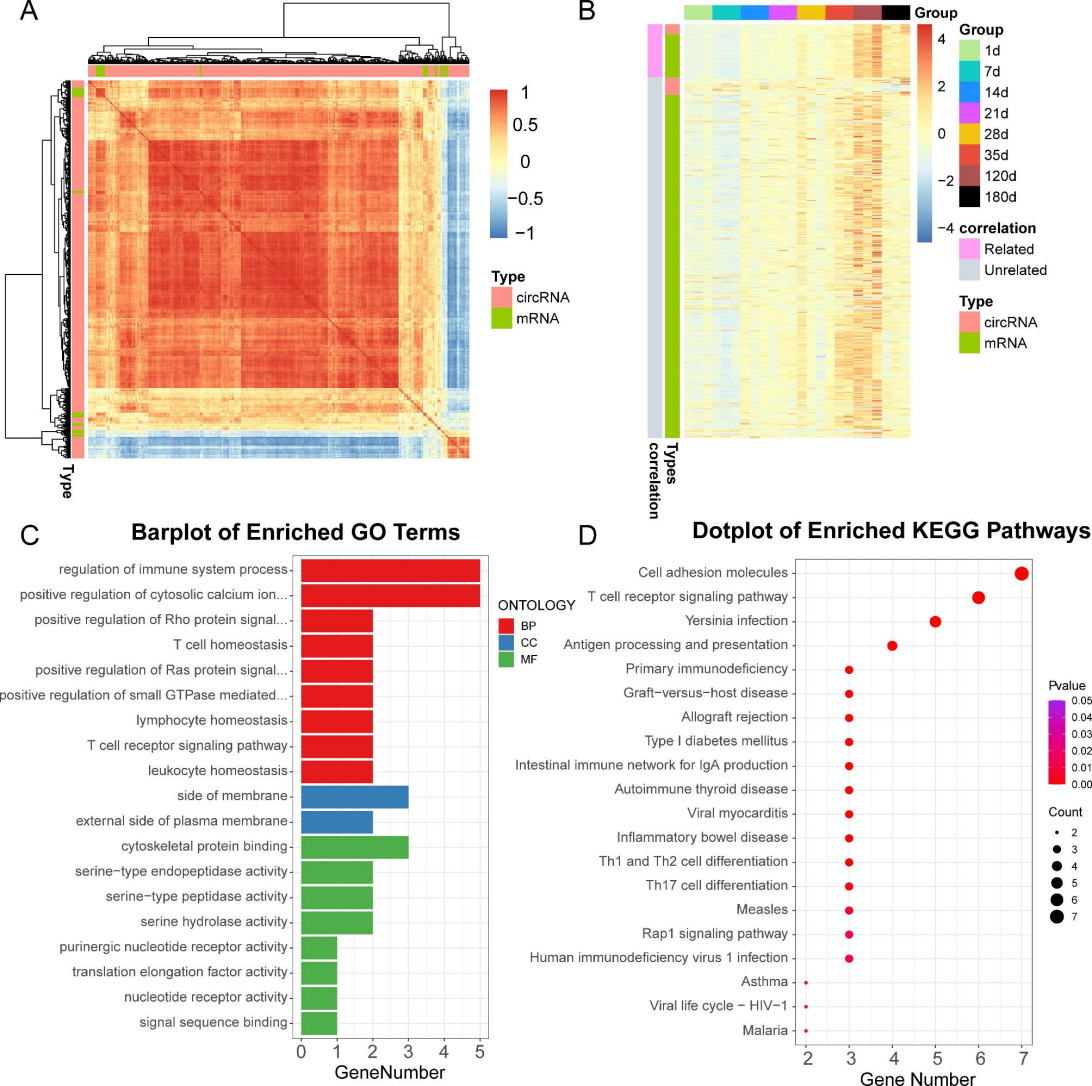
Correlation analysis and functional annotation of circRNA and mRNA expression profiles. (**A**) Correlation analysis of the circRNAs/mRNAs co-expression profile. (**B**) Heatmap of circRNA and mRNA gene expression profiles. (**C**) GO functional analysis of mRNAs associated with the DE circRNAs. (**D**) KEGG network analysis of mRNAs associated with the DE circRNAs.

Based on the above results, we identified 36 circRNAs in the red module, and 508 mRNAs in the darkturquoise module. We performed correlation analysis on the circRNAs/mRNAs co-expression (Fig. 5A). Our results revealed 13 circRNAs and 54 mRNAs that were strongly associated in their expression profiles, carrying a Pearson correlation coefficient > 0.85 (Fig. 5B, Table S5). We next conducted GO terms and KEGG network annotation analyses of the circRNA-associated genes. Using GO analysis, we revealed that the associated genes participated in immune regulation (Fig. 5C, Table S6). Using KEGG network analysis, we revealed that the T cell receptor signaling pathway, antigen processing and presentation, primary immunodeficiency, intestinal immune network for IgA production were the most immune-associated networks (Fig. 5D, Table S7).

### Generation of a possible circRNA-miRNA-mRNA modulatory axis

**Fig. 6:**
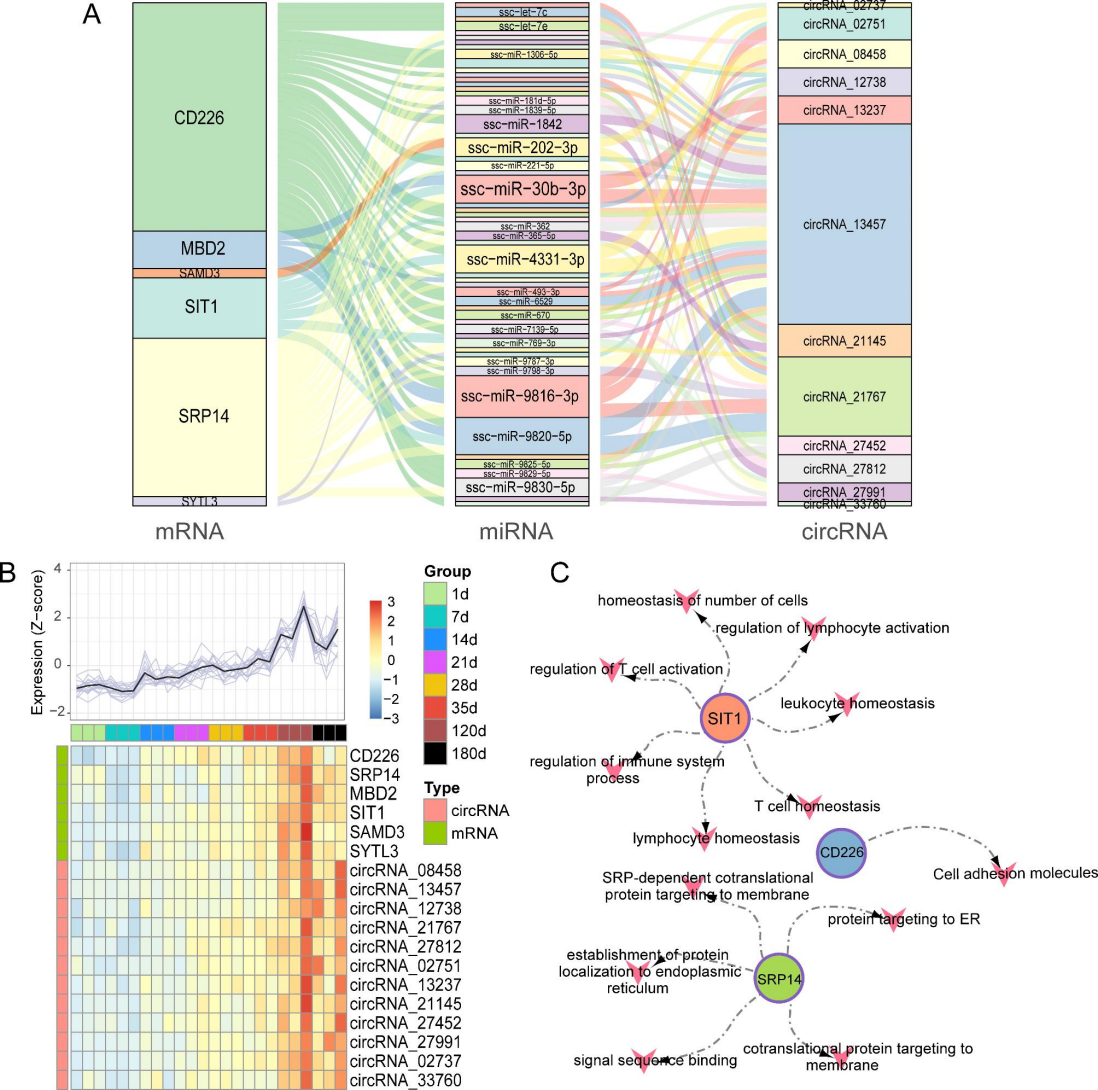
Generation of a potential circRNA-miRNA-mRNA modulatory axis. (**A**) Sankey plot decpiting the ceRNA axis estimated by the targeted associaiton and co-expression of circRNA-miRNA and miRNA-mRNA. Individual rectangles represent a single gene, and the connection degrees of individual genes are visualized according to the rectangular size. (**B**) Heat-map depicting the pattern of gene and circRNAs expression within the co-expression network. (**C**) Functional annotation of major genes within the co-expression network

**Fig. 7:**
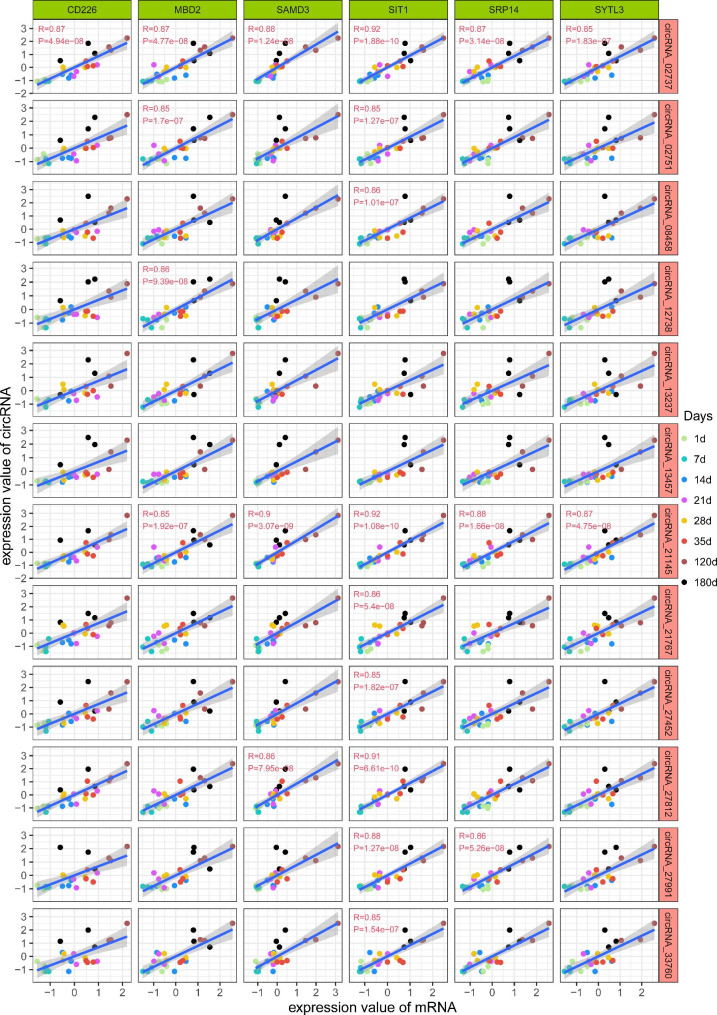
Scatter plot of circRNAs and mRNAs correlation analysis during Meishan pig spleen development. Individual genes and circRNAs depicting various levels of correlation. R refers to the correlation coefficient; *P* < 0.01 denotes significant correlation

To further elucidate the influence of circRNAs on mRNAs, in presence of miRNAs, during Meishan pig spleen development, we generated a ceRNA network using the aforementioned data. Table S8 illustrates the miRNA target gene prediction via the miRanda and Pita software. In all, we utilized 12 circRNAs, 24 miRNAs, and 6 mRNAs to generate the ceRNA network (Fig. 6A). Subsequently, we employed a heatmap to illustrate the expression profiles of *CD226, MBD2, SAMD3, SIT1, SRP14, SYTL3*, and their co-expressed circRNAs, with a rising trend over increasing developmental stage (Fig. 6B). We also screened for the major immune-related spleen networks, and discovered the *SIT1* gene to be a marked contributor of immune regulation (Fig. 6C). Additional correlation analysis revealed that all 9 circRNA expressions were strongly and directly associated with *SIT1* expression (Fig. 7). Collectively, these results suggested that circRNAs were DE across all 8 analyzed stages of porcine spleen development. Therefore, they can potentially modulate spleen development via regulation of host gene expression.

### Experimental verification of the circRNA-miRNA-mRNA regulatory network

**Fig. 8:**
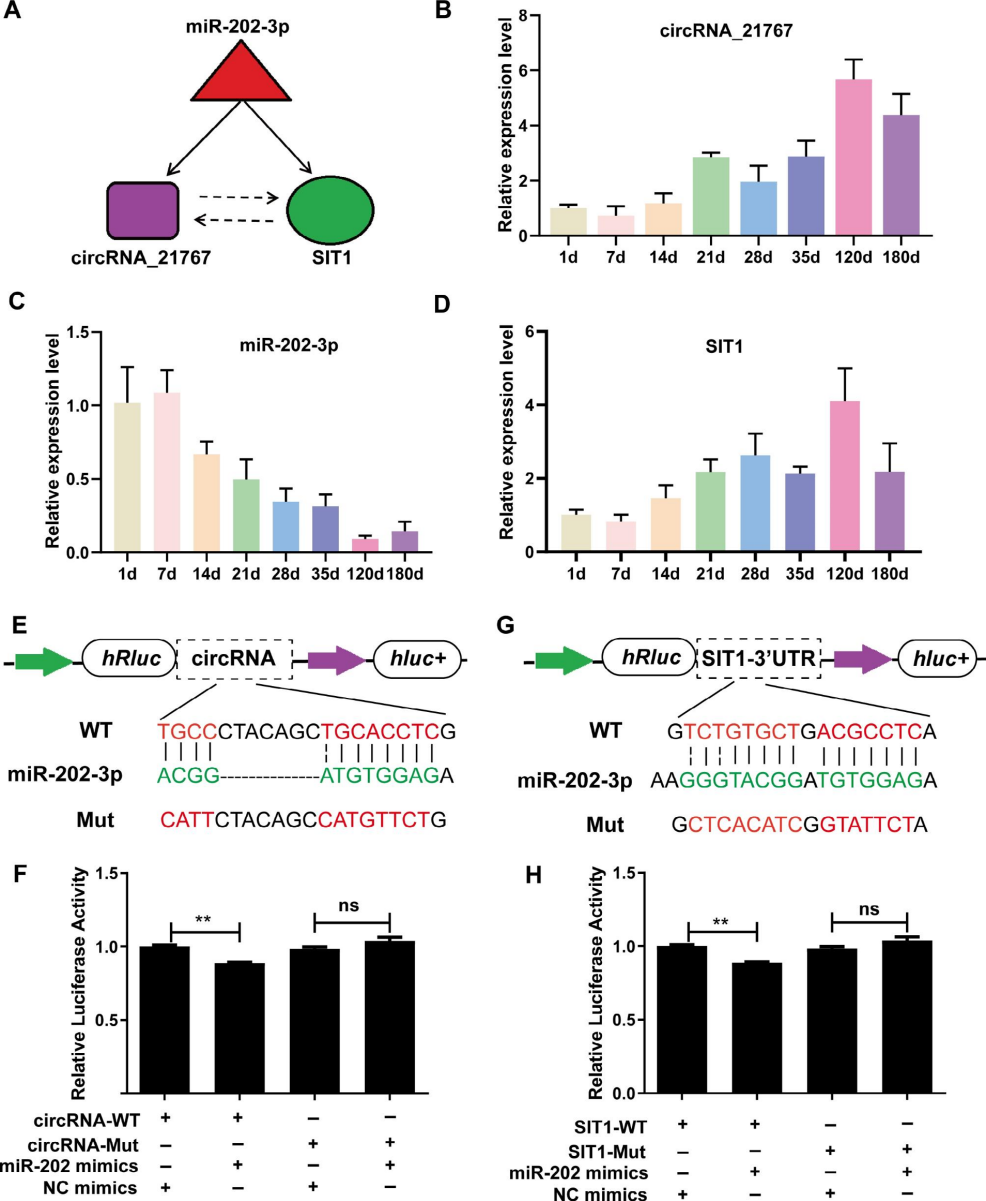
Experimental validation of circRNA-miRNA-mRNA regulatory circuitry. (**A**) Illustration of a three nodes circuitry including miR-202-3p, circRNA_21767 and *SIT1*. RT-qPCR shows the relative expression level of circRNA_21767 (**B**), miR-202-3p (**C**) and *SIT1* (**D**) during pig spleen development. (**E**) Predicted miR-202-3p binding site on circRNA_21767. The design of luciferase reporter. WT, the WT sequence of circRNA_21767 contains miR-202-3p binding site; Mut, the sequence of circRNA_21767 with mutation in miR-202-3p binding site. (**F**) PK15 cells were co-transfected with wild-type (WT) or mutant (MUT) luciferase reporters of circRNA_21767 with miR-202-3p mimics or negative control (NC) mimics. The relative levels of firefly luminescence normalized to Renilla luminescence are plotted. (**G-H**) Predicted miR-30a-3p binding site on *SIT1*-3’UTR. PK15 cells were co-transfected with wild-type (WT) or mutant (MUT) luciferase reporters of *SIT1* with miR-202-3p mimics, NC mimics. All data are presented as the mean ± SD, ^**^P < 0.01, ^*ns*^*P* > 0.05

Emerging evidences suggested that a series of sub-graphs containing 3 nodes modulate several biological functions [21]. Using circRNA network analysis, we identified a regulatory network with circRNA_21767, miR-202-3p, and *SIT1* (Fig. 8A). To confirm this network, we further evaluated the circRNA_21767 and *SIT1* contents using RT-qPCR at different points of spleen development. We demonstrated a close association between circRNA_21767 and *SIT1* expressions, suggesting that circRNA_21767 potentially modulated *SIT1* expression (Fig. 8B C). To further validate the association between miR-202-3p, circRNA_21767 and *SIT1*, we identified the potential miR-202-3p docking site within circRNA_21767 and 3’UTR of *SIT1* (Fig. 8D-F), using dual luciferase reporter assay. We demonstrated that miR-202-3p strongly suppressed circRNA_21767 luciferase activity. Moreover, miR-202-3p considerably diminished *SIT1* luciferase activity (Fig. 8G-H), relative to NCs (miR-NC and miR-202-3p inhibitor). Based on these evidences, circRNA_21767 potentially served as an miR-202-3p sponge to regulate *SIT1* expression. Taken together, these results revealed that three nodes circRNA mediated regulatory circuitry might play an essential role during spleen development in Meishan pigs.

### RNAseqTools construction for the visualization of transcriptome data

To assist biomedical researchers who lack programming expertise in analyzing and visualizing transcriptome sequencing data, we developed a user-friendly software named RNAseqTools (https://github.com/ChaoXu1997/RNAseqTool) using the Shiny framework (Fig. S2A). This interactive toolkit provides diverse analytical functions and visualization modes, such as, PCA, multi-group DE analysis, gene functional annotation, volcano plot, GSEA, gene trend analysis, and WGCNA (Fig. S2B). Its interactive visual interface will allow researchers to effortlessly elucidate data characteristics and distribution for basic research purposes.

## Discussion

Genome-wide analyses of RNA-seq data have revealed the abundance of circRNAs in animal transcriptomes and identified thousands of circRNAs in various species including humans, mice, livestock, and poultry, et al. [22]. CircRNAs have been identified in large numbers by deep RNA sequencing and are believed to be widely expressed in eukaryotic cells [23]. These findings suggest that there is still a considerable number of circRNAs that have not been discovered. Recent advancements in transcription profiling technology facilitates our growing understanding of non-coding RNA (ncRNA) and its immunomodulatory function. Investigations involving various animal species reported that circRNAs strongly modulate body’s normal immunity and immune imbalance, which is intricately linked to the development and progression of multiple immune diseases [24]. Moreover, circRNAs is also reported to function in a tissue- and pathogenesis-specific manner in many diseases [25]. Given its highly regulated profile of T and B lymphocytes, the spleen is the most essential organ for combating bacterial and fungul infections [18, 26]. Recent reports revealed the circRNAs characteristics across many tissues in pigs [19], as well as the DE gene and long ncRNA expressions during postnatal spleen development [27]. However, the identities and expression profiles of *S. scrofa* circRNAs remain largely uncharacterized in spleen tissue.

Spatio-temporal gene expression patterns elucidate the functions of genes within specific tissues or cells at particular times throughout the process of development. Existing research demonstrates the spatio-temporal regulation of circRNA expression in Drosophila brains [28], pig brains [16], and mouse pre-implantation embryos [29]. Additionally, circRNAs exhibit tissue-specific expression in the human heart and lung during fetal development [30]. Due to their precise spatio-temporal expression, it is essential that studies of circRNAs in mammals assess various tissues and developmental stages. Comprehensive examination of the spatio-temporal distribution of circRNAs is crucial for understanding their biological functions. The local pig breeds in China have underdone selective breeding for many generations, and have retained genetic diversities in existing pig populations. Hence, the domestic pig is an excellent model for spleen developmental study in mammals [31]. Herein, we performed genome-wide analysis of circRNA in spleen tissue at multiple developmental stages (from birth to adult) from Meishan pigs (a native Chinese pig), and our screening uncovered 39,641 circRNAs in the developing spleen of Chinese indigenous Meishan pigs. Based on our preliminary literature screening, this report is the first to extensively explore the circRNAs expression profile during the various developmental stages of pig spleen using RNA-seq data. Therefore, the conclusions of this study will be highly beneficial in the exploration and elucidation of circRNAs functions during spleen development. Our findings will also provide a theoretical basis for future investigations on human spleen tissue development and dysfunction.

CircRNAs are known to modulate numerous physiological processes using distinct signaling networks [32]. Although the molecular functions of circRNAs are mostly unclear, some circRNAs affect gene expression by acting as microRNA sponges. For example, CDR1as functions as an efficient miRNA sponge, binding miR-7 and allowing mRNAs to escape degradation following miRNA binding [33]. Besides, circRNAs serve as miRNAs “sponges” to control pig muscle development [13, 15, 34, 35]. However, its role in ceRNA regulation during spleen development remains unclear. Herein, we demonstrated that *CD226, MBD2, SAMD3, SIT1, SRP14*, and *SYTL3* were the strong modulators of spleen development, and these genes were further regulated by bundles of circRNAs. Subsequently, using KEGG analyses, we revealed that the cell adhesion molecules (CAMs) and immunomodulation of several genes were significantly enriched. Cell adhesion molecules, which express on the cell surface, facilitate activities of several biological processes, including, immune response, inflammation, and embryogenesis [36]. CD226 is exclusively expressed on the surface of Th1 cells, and they modulate Th1 differentiation [37]. Herein, we revealed that *SIT1* was strongly modulated by the circRNA_21767/miR-202-3p axis. Based on a prior investigation, signaling threshold regulating transmembrane adaptor 1 (SIT1), encoded by the *SIT1* gene, is a disulfide-linked homodimeric glycoprotein that interacts with the lymphocyte-specific transmembrane adaptor protein family to modulate the immunologic process [38]. It is implied that these genes can act as checkpoints to activate the immune response in spleen, and inhibitors of these genes (such as miRNA or circRNA) may be used for new non-antibody immune checkpoint inhibitors. The findings from this report provide novel evidence regarding circRNAs involvement in pig spleen immunity. Even though our software-simulated conclusions require further validation in vivo, our results confirmed that circRNAs indeed serve as miRNA sponges to modulate immune-associated gene expression within the spleen. Meanwhile, the non-coding RNA results from transcriptome analysis can serve as beginning experimental targets future in vivo verifications in animal models.

## Conclusion

In conclusion, this study presented the first reported catalog of circRNAs expression profile in the spleen tissues from Meishan pigs. We characterized numerous DE circRNAs that were relevant to the porcine spleen development. We also generated a comprehensive expression profile of various RNAs, particularly mRNAs and circRNAs, which, together, modulate a myriad of physiological processes within the Meishan pig spleen. Moreover, we identified a ceRNA network involving circRNA_21767/miR-202-3p/*SIT1*, which may modulate immune activities. Our findings provide candidate ncRNA-based targets for future in vivo animal study on the molecular regulation of immune function within the porcine spleen. Finally, we designed a user-friendly software named RNAseqTools that is available for use for the visualization of transcriptomic data using the Shiny app (https://github.com/ChaoXu1997/RNAseqTool).

## Materials and methods

### Animal treatment and sample collection

Overall, 24 healthy Meishan pigs were acquired from the Kunshan Conservation Ltd. (Suzhou City, Jiangsu Province, China) (Permission No. JS-C-05). Our examination involved 8 developmental stages, namely, 1d, 7d, 14d, 21d, 28d, 35d, 120d, and 180d after birth. All piglets were provided with the same standard diet, with housing at an environmentally regulated facility. Three piglets were sacrificed per treatment via an intravenous administration of pentobarbital sodium to minimize suffering. The spleen was immediately extracted and frozen in liquid nitrogen. Our animal care and treatment protocols were approved by the Institutional Animal Care and Use Committee (IACUC) of Yangzhou University (No. SYXK(Su)2021-0026).

### RNA extraction, quality assessment and library construction

Splenic total RNA extraction employed TRIZOL (Invitrogen, Carlsbad, CA, United States) following kit directions. RNA quality assessment employed an Agilent 2100 Bioanalyzer (Agilent Technologies, Palo Alto, CA, United States). Ribosomal RNA elimination from extracted RNA utilized the Ribozero™ rRNA Removal Kit (Epicenter, United States). Subsequently, linear RNA was eliminated via an RNAse R kit (Epicenter, United States). Lastly, sequencing libraries were generated with rRNA-free and linear RNA-free RNA using the NEBNext® Ultra™ Directional RNA Library Prep Kit (NEB, Ipswich, MA, United States).

### CircRNA sequencing and screening

Sequencing of generated libraries was done via the Illumina HiSeq 2500 platform (Oebiotech Corporation, Shanghai, China). TopHat2 (v2.1.1) mapped the clear data against the porcine reference genome (*Sscrofa11.1*). We uploaded the resulting RNA-seq information in the Gene Expression Omnibus (accession codes GSE228936). To construct the SAM file, we employed BWA [39] for alignment of the sequencing reads of individual samples to the porcine genome. Lastly, we identified circRNA using CIRI2 tools (version 1.2) [40]. The circRNA sequence estimation employed junction reads and GT-AG cleavage signals. The identified circRNAs were further assessed according to their type, chromosome distribution, and length distribution using find_circ (version 1) [41] and the annotation information of the *Sscrofa11.1* genome.

### Analysis of the differentially expressed (DE) circRNAs

DE circRNAs were identified using the DESeq2 package [42], based on the following criteria: absolute fold change ≥ 2 and adjusted *p*-value < 0.05.

### Generation of WGCNA

We generated a co-expression network across all examined developmental stages using the R package WGCNA [43]. This allowed us to further analyze protein-coding genes and circRNAs sets that were critical at each stage of development.

### Establishment of the circRNA–miRNA–mRNA ceRNA axis

We retrieved the miRNA sequences from the miRbase (https://mirbase.org/, accessed on 20 December 2022) website, and the mRNA 3’UTR sequences from BioMart (https://asia.ensembl.org/index.html, downloaded on 25 December 2022) [44]. To explore the associations and functions of various ncRNAs and mRNAs, we next generated an ncRNA–mRNA modulatory axis. MiRanda (version:3.3a) [45] predicted the circRNA–miRNA–mRNA pairs (score threshold ≥ 150 and energy threshold ≤ − 20). The circRNA–miRNA–mRNA co-expression axis was then generated with the Cytoscape software (v3.2.1) [46] to examine the role of major circRNAs. Finally, we visualized the network using the ggalluvial R package (Version: 0.9.1).

### Functional enrichment analysis (FEA)

We performed the GO term and KEGG pathway [47–49] enrichment analyses of circRNA-associated genes using the R package clusterProfiler [50], using the following criteria: corrected P-value < 0.05 was the significance threshold.

### Quantitative RT-PCR (qPCR) validation

qPCR employed an Applied Biosystems qPCR apparatus and SYBR green master mix (Vazyme, Nanjing, China), as per kit protocols. The reaction mixture was composed of 5 µl SYBR Green Mixture (Vazyme, Nanjing, China), 1 µl cDNA template, 0.2 µl primer (each), and 3.6 µl deionised water, and the conditions were set as follows: 95 °C for 5 min, 40 cycles of 95 °C for 10 s, and 60 °C for 30 s. All employed primer sequences were summarized in Table S1. *GAPDH* (for mRNA and circRNA) and *U6* (for miRNA) served as controls for normalization purpose, and all qRT-PCR reactions were performed three times, and the average adjusted value was used for analysis. Relative gene expressions were computed using the 2^−ΔΔCt^ formula. All data analysis employed the unpaired 2-tailed Student’s t-tests; ^*^p < 0.05, ^**^p < 0.01, and data are provided as mean ± SD (standard error of mean).

### Dual-luciferase reporter assays

We inserted wild-type (WT) or mutant-type (Mut) *SIT1*-3’UTR or circRNA_21767 downstream of the pmirGLO Dual-Luciferase vector. Next, we plated cells into a 24-well plates, and co-transfected the cells with the WT (*SIT1*-WT or circRNA_21767-WT), or Mut (*SIT1*-Mut, circRNA_21767-Mut), and miR-202-3p-mimics (NC-mimics). Finally, we detected both firefly and renilla bioluminescence using the DLR Gene Assay Kit (Beyotime, Shanghai, China). The firefly luciferase activity was then adjusted with the control renilla luciferase activity for a measurement of the constructed reporter luciferase activity.

*SIT1*-WT:

TCCTCCCAACCCCCAAACTCCCAGGTTTTCAGTCCTCCTTCCGGAGTTTAATCAGATGCTCCCCACTCCGGCTGCCTCATGG**TCTGTGCTGACGCCTC**AGTGTCTCCTCAGCCACAGGAAGTAGGCAGTGGGGGAGGGGGTTAGAGCCTGAGAGGATATGTATGGGTATC.

*SIT1*-Mut:

TCCTCCCAACCCCCAAACTCCCAGGTTTTCAGTCCTCCTTCCGGAGTTTAATCAGATGCTCCCCACTCCGGCTGCCTCATGG**CTCACATCGGTATTCT**AGTGTCTCCTCAGCCACAGGAAGTAGGCAGTGGGGGAGGGGGTTAGAGCCTGAGAGGATATGTATGGGTATC.

circRNA_21767-WT:

TAGTCTTGTATTGATGGTTTTGCACTATTTACAGAccctACCTGAAcctatccttccatccatccaaaAAATGTA**CCCA**GAATCTTTTAGTCC**TGCC**CTACAGC**TGCACCTC**GTACATCAAGCTCCATATAATGTTCCTCCTTACCTCTCAAAGAACGAATCAAATCTTGGGGACCTCTTAT.

circRNA_21767-Mut:

TAGTCTTGTATTGATGGTTTTGCACTATTTACAGAccctACCTGAAcctatccttccatccatccaaaAAATGTA**TTTG**GAATCTTTTAGTCC**CATT**CTACAGC**CATGTTCT**GTACATCAAGCTCCATATAATGTTCCTCCTTACCTCTCAAAGAACGAATCAAATCTTGGGGACCTCTTAT.

### Electronic supplementary material

Below is the link to the electronic supplementary material.

Supplementary Material 1

Supplementary Material 2

## Data Availability

The circRNA sequencing datasets during the current study are available in the Gene Expression Omnibus repository (accession codes GSE228936).
