# Supplementary material for: Genome-wide analysis of circRNA regulation during spleen development of Chinese indigenous breed Meishan pigs
Source: BMC Genomics. 2023 Aug 23;24:477. doi: 10.1186/s12864-023-09612-x (PMC10463621; doi:10.1186/s12864-023-09612-x)
Supplement: Supplementary file 1 — Supplementary Material 1 [file 12864_2023_9612_MOESM1_ESM.docx]

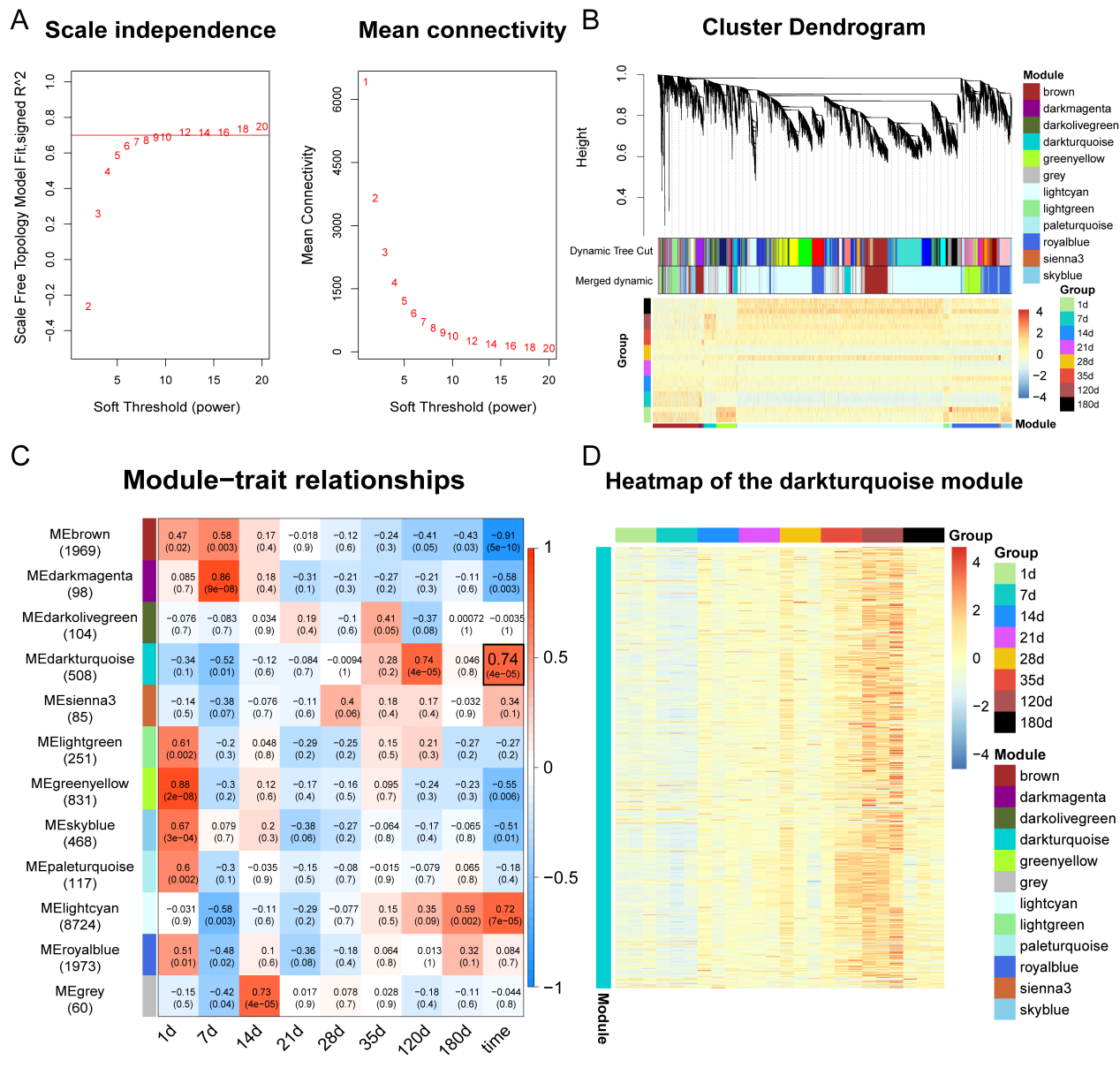


**Fig. S1. WGCNA analysis of mRNAs dataset identified** **co-expression module in spleen development of Meishan pigs**. (A) Scale independence and mean connectivity analysis for various soft threshold powers. (B) Clustering dendrograms of pig spleen samples in different time points. 12 modules represented by colors in the horizontal bar are found using 0.25 threshold merging. (C) Module–trait relationship. (D) Heatmap showing the mRNA expression pattern of darkturquoise module.


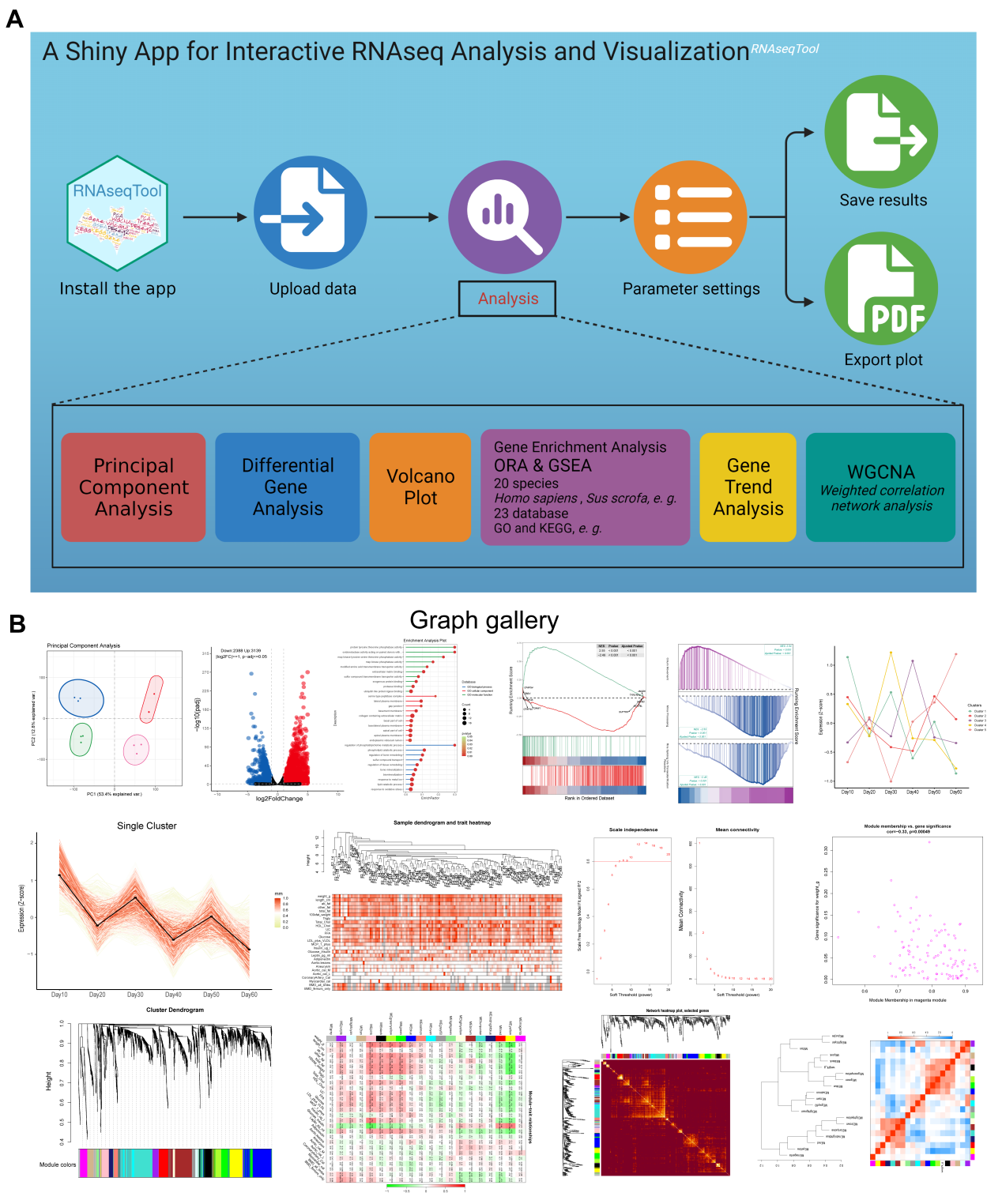


**Fig. S2. Overview of core features and analysis in the RNAseqTool shiny app.** (A) The user-friendly, interactive Shiny app can analyze transcriptome sequencing data, including various analysis and visualization functions. (B) Graph gallery shows example outputs of RNAseqTool, and more example outputs can be viewed directly by installing the software.
